# Effects of constructing farmland with large amounts of iron tailings as soil reconstruction materials on soil properties and crop growth

**DOI:** 10.1038/s41598-022-24599-3

**Published:** 2022-11-23

**Authors:** Wenjuan Jin, Zhongyi Wei, Xinzheng Liu, Qi Li, Chunlan Han, Zhenxing Bian, Xufeng Zhang, Fengkui Qian, Yonghai Liu

**Affiliations:** 1 College of Land and Environment, Shenyang Agricultural University, Shenyang, 110161 China; 2 Key Laboratory of Trinity Protection and Monitoring of Cultivated Land, Shenyang, 110161 China; 3 Liaoning Natural Resources Affairs Service Center, Shenyang, 110011 China; 4 Jianping Shengde Rixin Mining Co., Ltd, Chaoyang, 122400 China

**Keywords:** Environmental sciences, Environmental impact

## Abstract

With continuous population growth and farmland decrease, the food security is seriously threatened. Farmland reclamation has been used as a means of raising the agricultural productivity and improving the ecological environment. However, the lack of reclaimed soil represents a serious problem. To verify the feasibility and effect of using large amounts of iron tailings to construct farmland, ten treatments (T1–T10) were designed to represent different soil profiles of regional normal farmland and constructed profiles using iron tailings. All treatments involving an iron tailings layer below topsoil exhibited higher soil water contents. The field capacity under T3 (20-cm iron tailings layer below cinnamon soil (b)) was 19.20% higher than that under T7 (20-cm red clay layer below cinnamon soil (b)), and the field capacity under T5 (20-cm iron tailings layer below cinnamon soil (a)) was 2.26% higher than that under T9 (20-cm red clay layer below cinnamon soil (a)). The soil water contents under T3 and T5 were almost the same as those under T7 and T9, respectively. The water-holding capacity of the 30-cm iron tailings layer (T6) was better than that of the 20-cm iron tailings layer (T2). Additionally, none of the treatments caused salt injury to maize. The maize height and stem thickness under the treatments employing iron tailings layers below topsoil were significantly greater than those in normal farmland; the maize height and stem thickness under T3 were 136.82% and 32.02% greater, respectively, than those under T7, and the values under T5 were 9.13% and 9.56% greater, respectively, than those under T9. The maize yields matched or even surpassed those in normal farmland, namely, the maize yield under T5 was equal to that under T9, and the maize yield under T3 was 12.69% higher than that under T7. In general, the application of an iron tailings layer below topsoil to construct farmland is a feasible and environmentally friendly way to realize sustainable farmland utilization and is beneficial to soil quality and crop yield improvement. Collectively, these results provide insight into the efficient utilization of iron tailings and environmental protection.

## Introduction

Mining is an economic activity that intensified during the twentieth and twenty-first centuries to satisfy the demands of industrial development, which has caused serious social, environmental and economic impacts, especially in resource-rich regions^1,2^. China is one of the first countries worldwide to exploit and utilize iron ore, with abundant reserves and a high annual output. The increasing scale of mining inevitably causes large-scale land disturbance and severe damage to the vegetation, soil, and landscape of the original landform, posing a serious threat to the regional ecological security^3–5^. The conflict between human development and farmland protection in China has become increasingly aggravated^6,7^. Therefore, it is necessary to reclaim abandoned mines, construct farmland and accelerate ecological restoration^8,9^.

Land reclamation can be divided into five stages from the perspective of the corresponding workflow, including landform reshaping, soil reconstruction, vegetation reconstruction, landscape representation, and biodiversity reorganization and protection^10^. Among these stages, soil reconstruction constitutes the core component of land reclamation and ecological restoration in mining areas, and the quality of reconstructed soil directly determines the quality of land reclamation^10,11^. Soil reconstruction refers to the implementation of appropriate reconstruction techniques and application of engineering measures and physical, chemical, biological and ecological methods to construct appropriate soil profiles and soil fertility conditions for soil restoration purposes in industrial and mining areas. At the same time, it is necessary to stabilize the landscape, restore and improve the soil productivity rapidly, and improve the environmental quality of the reconstructed soil^11^. The materials used for soil reconstruction include both soil and the soil matrix and other mine waste materials, such as rock, gangue, fly ash, slag, low-grade ore, or a mixture of two or more of these materials^12^. The most ideal soil reconstruction material is the original soil stripped in the mining process, but it is difficult to obtain sufficient high-quality soil^13^. Especially in China, many historical legacy mines exist, and there is a serious lack of soil resources, which makes appropriate soil substitute materials vital to mine site reclamation.

Soil substitute materials, also referred to as artificial soil or new soil source materials^14^, involve a reasonable ratio of solid waste and other nontopsoil resource materials from the perspective of land reclamation and sustainable environmental development in mining areas to provide new soil suitable for plant growth^15^. The preferred soil substitute materials are generally industrial solid waste materials obtained from mining areas. Many laws and regulations concerning land reclamation in Western countries stipulate that it is necessary to analyze the physicochemical properties and heavy metal content in the soil matrix and overlying strata and subsequently select suitable soil substitute materials before mining^16,17^. For example, in Germany, there occur fewer topsoil sources, some of which include stripped topsoil in mining areas and artificial soil^18^. In the United States of America, it is necessary to identify and screen suitable soil substitute materials from the overburden strata of coal seams when the amount of stripped soil is insufficient. Baker et al*.*^19^ used coal gangue prepared from combustion products such as fly ash and sludge to produce stable soil that could be directly applied in farmland. Wilson and Skousen^20^ used unweathered gray sandstone and weathered brown sandstone in soil reconstruction of an open-pit mine in West Virginia, USA, and experimental observations over eight consecutive years revealed that weathered brown sandstone soil exhibited satisfactory physicochemical properties and was an ideal soil substitute material. In Indonesia, the utilization of fly ash as a soil substitute material in mining areas was achieved by mixing topsoil and fly ash based on the physical characteristics and boron amount eluted from fly ash^21^. The incorporation of eco-engineering tailings into soil-like growth media has been advocated as a promising technique for cost-effective and sustainable rehabilitation of iron tailings without the need for large volumes of natural topsoil^22^. However, few studies have considered iron tailings as a soil substitute material.

Research on soil substitute materials in China started relatively late. Regarding coal mining area reclamation, coal gangue^23,24^ and fly ash^25,26^ are usually selected as alternative materials for soil reconstruction. Through pot and plot experiments, Hu et al.^13^ used the overlying rock and soil layer in an open-pit coal mine in Inner Mongolia as a matrix and incorporated different additives to improve topsoil alternatives for dump reclamation, and the results demonstrated that Layer III of the matrix after weathering was more suitable as a topsoil substitute material. In addition, his team added peat^27^, modified straw^28^, vermiculite^29^, and humic acid^30^ and improved and optimized the formula of the applied soil substitute material by assessing alfalfa growth and resisting performance. Because of the different ore-forming geological conditions occurring in different regions, the physical and chemical properties of mining solid waste materials are different, so the proportion of solid waste materials exhibits notable regional characteristics. Hu et al.^31^, Duo and Hu^32^, and Zhao et al.^33^ proposed a new technology of using Yellow River sediments as reconstructed soil material to mitigate coal mining-induced subsidence. In regard to soil reconstruction during iron ore reclamation, Lu^34^ studied the physical and chemical properties of dry discharge iron tailings and corresponding reclamation technology and found that a grinding cultivation matrix covering a 30-cm iron tailings layer could represent a suitable reclamation mode. Fan^35^ used the pot test method to transform iron ore waste rocks and soil into reclamation mixed soil according to different mixing proportions. By analyzing the physical and chemical properties of the obtained mixed soil and its impact on maize growth, it was found that iron ore waste reclamation mixed soil at a 3:7 ratio was the most suitable for maize growth.

At present, waste iron tailings are mostly used as soil fertilizers worldwide, but their usage is limited, making it difficult to solve various problems, such as iron tailing accumulation and pollution. In northern China, large amounts of iron tailings produced during iron ore mining and selection are stored in tailings ponds and are often regarded as waste materials^36^. These tailings ponds not only involve a large amount of land occupation but also involve the need for passive tailings pond reclamation. There are many similar places in the world. Western Liaoning Province, a region with large amounts of iron tailings, belongs to the semiarid and typical ecologically fragile area, where the native topsoil is relatively infertile, and reclamation soil resources are seriously lacking, which has become a serious bottleneck in the land reclamation process in this area and has caused various problems, such as an insufficient soil cover thickness and poor vegetation growth. Wei and Liang^37^ innovatively proposed the idea of mixing iron tailings with topsoil to increase the soil layer thickness and improve medium- to low-yield fields in western Liaoning Province. It has also been demonstrated that the incorporation of an appropriate amount of iron tailings into topsoil can improve soil physical properties and crop yields and does not contaminate farmland soil^38^. In addition, Davila et al*.*^39^ and Almeida et al*.*^40^ evaluated productivity and metal accumulation in the Fundao dam area (Minas Gerais, Brazil), released large amounts of iron tailings and determined that the concentrations of metals remained within the limits allowed for human consumption, thus not posing long-term risks; they indicated that the deposited tailings would not cause future regional heavy metal contamination. The results of our previous study revealed that the contents of heavy metals such as Cd, Cr, Cu, Zn, Pb, Ni, Hg and As did not exceed the risk intervention values for farmland contamination in China^41^, and the heavy metal content was even lower than that in regional native soil, with no toxicity and no pollution sources for soil and crops^38^. Thus, constructing farmland with large amounts of iron tailings could represent a promising method for farmland reclamation.

Few studies regarding the notable use of iron tailings for farmland construction in mining areas have been reported to date. Therefore, our general aim was to demonstrate the feasibility of farmland construction involving large amounts of iron tailings. Via full utilization of common raw materials in the mining area, including topsoil, iron tailings, waste rocks and stripped soil, 10 types of farmland soil profiles were designed. Considering the semiarid climatic conditions in the study area, soil moisture characteristics were closely monitored. Additionally, crop growth conditions and yields were regularly monitored. The main objectives of this study were to (1) verify the feasibility of a new soil reconstruction material (iron tailings) that could be used to construct farmland and describe the associated reclamation technology and construction process and (2) to analyze soil properties and crop growth and yield after reclamation.

## Materials and methods

### Study area

**Figure 1:**
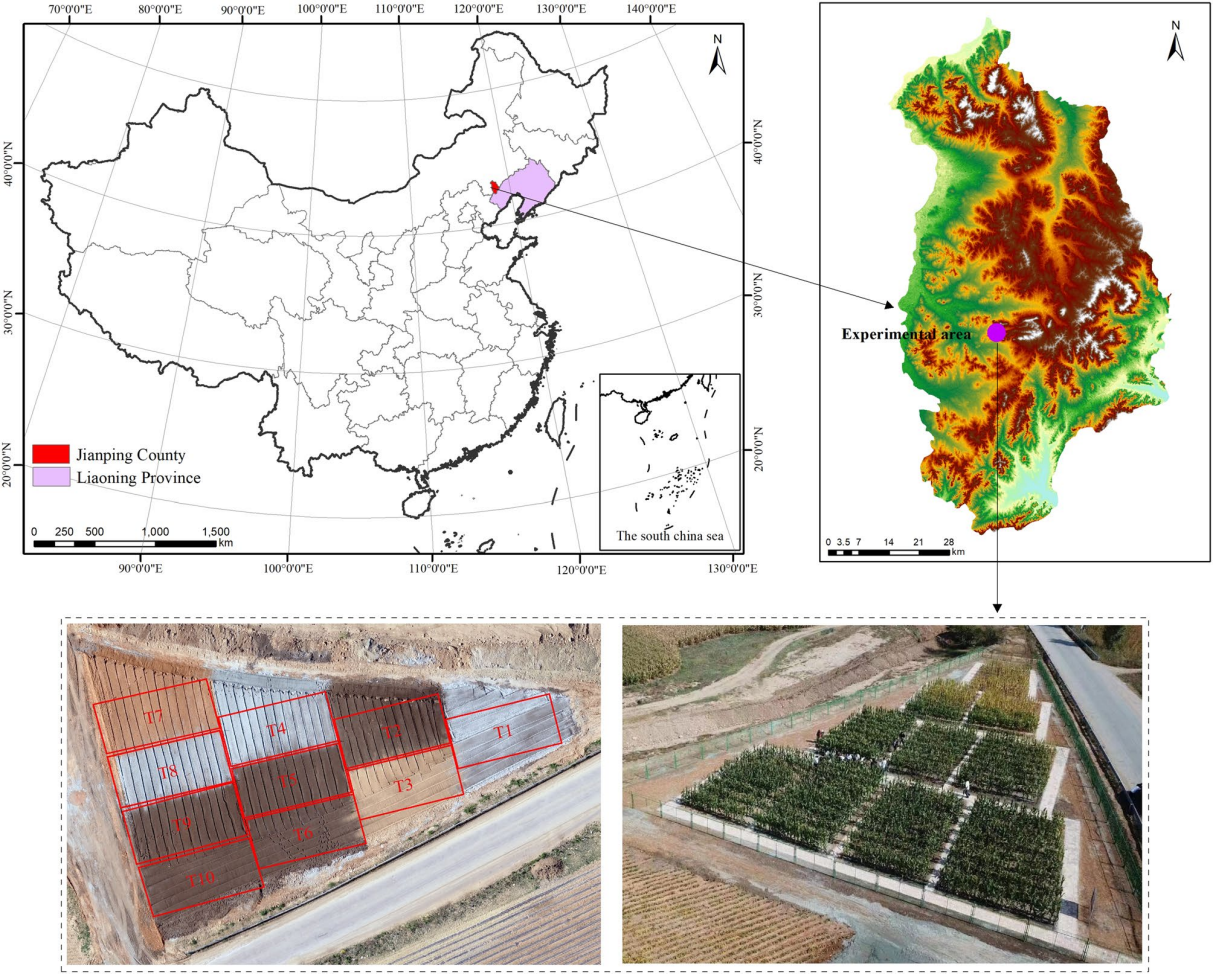
Location of the study area (the red rectangular boxes represent the distribution of the experimental plots of reclaimed farmland. T1 to T10 indicate the ten types of reclamation treatments. The map images were obtained from www.google.com/maps. The photos of the experimental site were taken during the field investigation).

The experimental site is located in a newly reclaimed farmland area in Xincheng village (41°45′ N, 119°37′ E), Xiaotang town, Jianping County, Chaoyang city, Liaoning Province, northern China (Fig. 1), which is an area rich in iron ore resources. Mining waste materials, such as iron tailings and waste rock materials, occupy large amounts of land. This area is characterized by a semiarid monsoonal climate with a mean annual temperature of 7.6 °C, a mean annual precipitation of 614.7 mm, and a mean annual effective evaporation of approximately 1853 mm. According to the soil classification system of China, the soil in this region belongs to Haplic-Ustic Argosols^42,43^. The original farmland in the region exhibits a thin tillage layer (approximately 15 cm), heavy clay soil texture, weak soil moisture regimes, and low soil organic matter content, which leads to the farmland in western Liaoning Province mostly providing medium- and low-yield fields. Aridity is the primary limiting factor of the regional farmland quality^44^. In addition, the thin topsoil layer leads to an extreme lack of soil resources for land reclamation.

### Reclamation procedures

**Figure 2:**
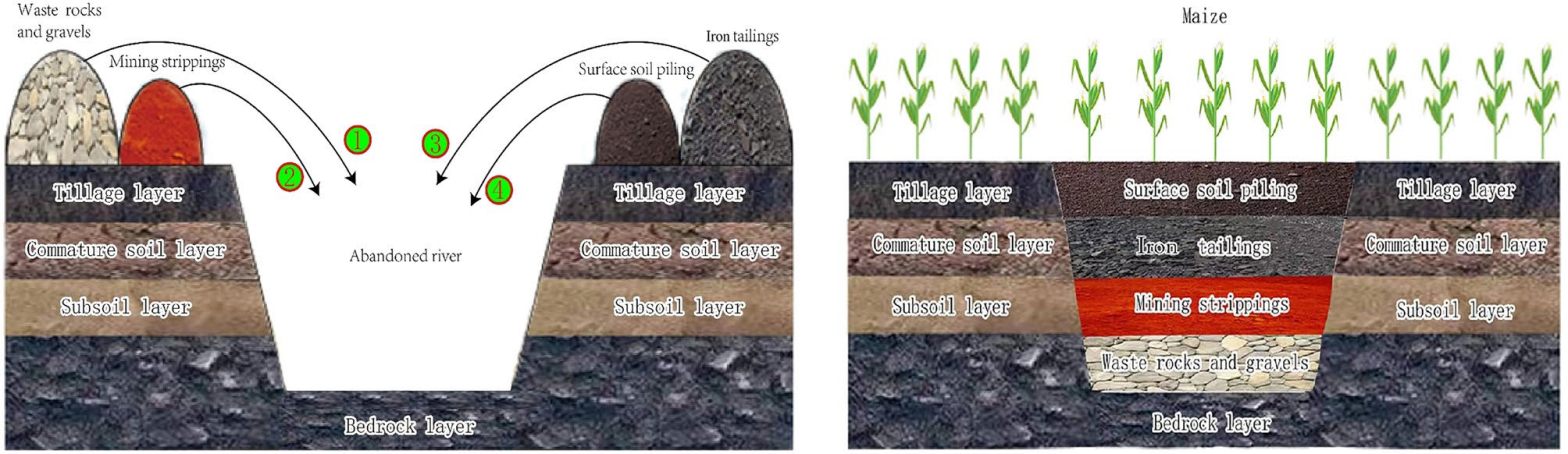
Photos of the farmland construction procedure (Note: ①, ②, ③, and ④ indicate the placement sequence of the structural materials; This image was drawn by the first author).

Combined with land construction planning, a section of the abandoned river in Xincheng village was selected for the experiment, and experimental site construction began in March 2020 and was completed in April 2020. The process of farmland construction involving large amounts of iron tailings was as follows (Fig. 2): first, waste rock materials stripped during mining were placed at the bottom to increase the ground elevation. Next, mining gravel was emplaced atop the waste rock materials as a foundation for cultivation construction. Then, subsoil stripped during mining was placed atop the gravel layer as an impermeable layer. Finally, iron tailings and stripped topsoil were emplaced layer by layer atop the subsoil layer. The total thickness of topsoil and iron tailings ranged from approximately 40 to 60 cm. Iron tailings are generally employed to increase the soil thickness of the tillage layer and serve as a water-holding layer in farmland.

**Figure 3:**
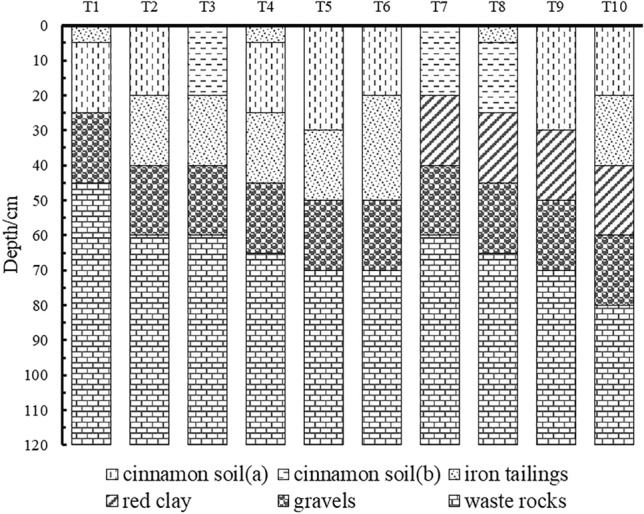
Schematic diagram of the soil profiles under the different farmland construction treatments.

Considering the specific local conditions, 10 experimental plots (12 × 6 m) were established combined with previous research results for iron tailings mixing into topsoil (Yang, 2016; Wei and Liang, 2016) (Fig. 1), representing 10 types of construction treatments (T1–T10) (Fig. 3). In each plot, three 6 × 4 m repeated test subzones were established. Waste rock and gravel materials were produced by stripping bedrock during mining. The lower layer stripped during pit excavation and mining, mainly referred to as red clay in this paper, is a relatively clayey soil layer occurring several meters to more than ten meters below the surface. Iron tailings were acquired from dry discharge iron tailings remaining after the iron mining activities of the Jianping Shengderixin Mining Co., Ltd., Chaoyang city, Liaoning Province. After testing, the contents of heavy metals such as Cu, Pb, Zn, Fe, Ni, Mn and Cr in the obtained iron tailings did not exceed the risk intervention values for agricultural land contamination in China^41^, and the heavy metal content was even lower than the environmental background value (the heavy metal content in regional native soil), with no toxicity and pollution risks to soil and crops. Topsoil was obtained from topsoil dumps formed via topsoil stripping during mining, exhibiting two colors, i.e., brown and yellow, but both these soils were cinnamon soil in the Chinese soil classification system^42,43^. Thus, these soils were named cinnamon soils (a) and (b), respectively. In addition, all the structural materials were stacked near the experimental site, and the essential traits of the red clay, iron tailings, and stripped topsoil are listed in Table 1. Under the T1 treatment, iron tailings were placed atop a 20-cm cinnamon soil (a) layer overlying the gravel layer. Under treatments T2, T3, T4, T5 and T6, a 20-cm or 30-cm iron tailings layer was placed atop the gravel layer to replace red clay as the water-retention layer, and topsoil layers of different types and thicknesses were employed. Under the T7, T8, and T9 treatments, a 20-cm red clay layer was placed atop the gravel layer, followed by topsoil, representing the normal farmland soil profile in the study area. Under treatment T10, a 20-cm red clay layer was placed atop the gravel layer, and iron tailings and topsoil were then emplaced layer by layer atop the red clay layer. In addition, the previous research results of our research group regarding the application of a 5-cm mixed iron tailings-topsoil layer to improve the soil quality were applied to treatments T1, T4 and T8 to further verify these findings.

**Table 1:** Essential characteristics of the practical materials.

Practical materials	Mechanical composition (%)			Texture	Bulk density (g·cm^−3^)	Porosity (%)	Moisture content (%)	EC (S·m^−1^)	Organic carbon (g·kg^−1^)
	Clay (< 0.002 mm)	Silt (0.05 ~ 0.002 mm)	Sand (> 0.05 mm)						
Red clay	49.55	24.98	25.47	Clay	1.49	59.51	58.5	0.36	1.71
Iron tailings	0.23	31.28	68.42	Sandy loam	1.53	44.2	8.9	0.17	6.09
Cinnamon soil (a)	32.88	47.82	19.30	Silty clay loam	1.39	53.3	17.1	0.27	16.16
Cinnamon soil (b)	31.73	49.19	19.08	Silty clay loam	1.41	53.9	17.5	0.25	15.88

### Planting and management

After reclamation, each plot was covered with mulch, and maize (*Zea mays* L., i.e., Gaoke 985) was planted in each experimental plot according to the national guidelines for maize cultivation. The planting density was 60,000 plants per hectare. The seeding depth ranged from approximately 3 to 5 cm. Before seeding, 450 kg of stable composite fertilizer (the effective content of N-P_2_O_5_-K_2_O = 25-11-12%) was applied per hectare as base fertilizer. All the fertilizer was first evenly broadcast across the surface and then incorporated with a rotary tiller. Drip irrigation pipes were laid in the experimental plots, and supplementary irrigation was performed via drip irrigation.

### Soil sampling and analysis

The main purpose of this paper was to explore the feasibility of the application of large amounts of iron tailings to construct farmland, thereby improving regional medium- and low-yield fields and increasing the crop yield. Considering the semiarid climate conditions in western Liaoning Province, the bulk density, field capacity, soil water content, soil electrical conductivity (EC), and crop growth and yield under the different treatments were monitored. The bulk density reflects the effect of iron tailings on improving the local clayey soil texture. The feasibility of replacing soil with iron tailings as a water-retaining layer to obtain reclaimed soil was reflected by soil moisture indicators, and the feasibility of using iron tailings to increase crop yields was characterized by crop growth and yield indicators.

Soil and crop samples were collected in July 2020 and October 2020, and during each sampling, the ten experimental plots were sampled. Samples were obtained from the topsoil layer (0–20 cm). Both the bulk density and field capacity were measured via the cutting ring method^45^. Both the soil water content and soil EC were measured with a moisture teller (PICO-BT 0809)^46–48^. The plant height and stem thickness of maize were measured once every 5 days during the growing period using a standard meter and Vernier caliper, respectively. The dry weight and maize yield were measured at harvest to determine the grain yield of 30 randomly selected plants in each experimental plot, which was converted into the per hectare yield.

### Data analysis

The experimental data were first processed in Excel 2016 (Microsoft Corp., Redmond, WA, USA), and the STDEVP function in Excel 2016 was used to calculate the standard deviation (SD) of each treatment. Then, the experimental data were analyzed using statistical software SPSS 25 (SPSS Inc., Chicago, IL, USA)^49^. One-way analysis of variance (ANOVA) followed by Tukey’s test (P < 0.05) was used to calculate the significant difference in soil physical indicators among the different treatments. Origin 2017 was used to graph and present the data.

## Results

### Soil properties

#### Bulk density

**Figure 4:**
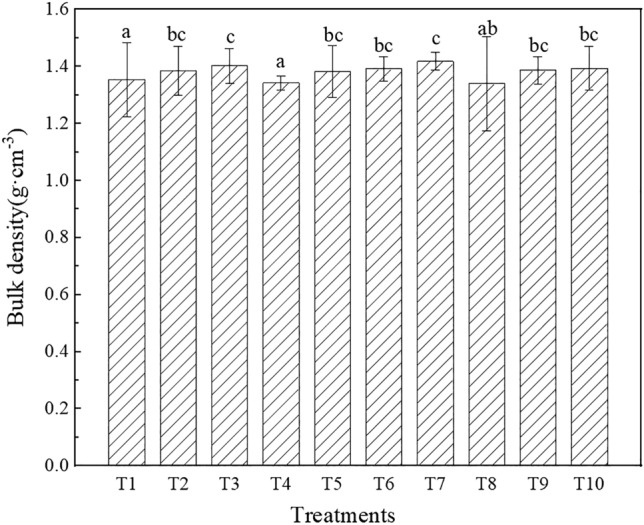
Bulk density under the different treatments (the different letters denote significant differences at the 0.05 level).

The bulk density under the different treatments is shown in Fig. 4. The bulk density significantly differed among the different types of topsoil, but no significant differences in the remaining indices were noted among the various topsoil types (P < 0.05). The bulk density ranged from 1.34 to 1.40 g·cm^−3^ and was ranked as T8 < T4 < T1 < T5 < T2 < T9 < T6 < T10 < T3 < T7. Among all treatments, a 5-cm mixed iron tailings-topsoil layer was employed under T1, T4 and T8, and the bulk density was lower than that under the other treatments. The topsoil under the T2, T5, T6, T8 and T10 treatments was cinnamon soil (a), and the bulk density was lower than that under the T3 and T7 treatments, whose topsoil comprised cinnamon soil (b). The bulk density under T8 was 5.50% lower than that under T7, and the value under T4 was 3.08% lower than that under T2. Mixing iron tailings into topsoil could significantly reduce the bulk density. The bulk density of cinnamon soil (a) was lower than that of cinnamon soil (b).

#### Field capacity

**Figure 5:**
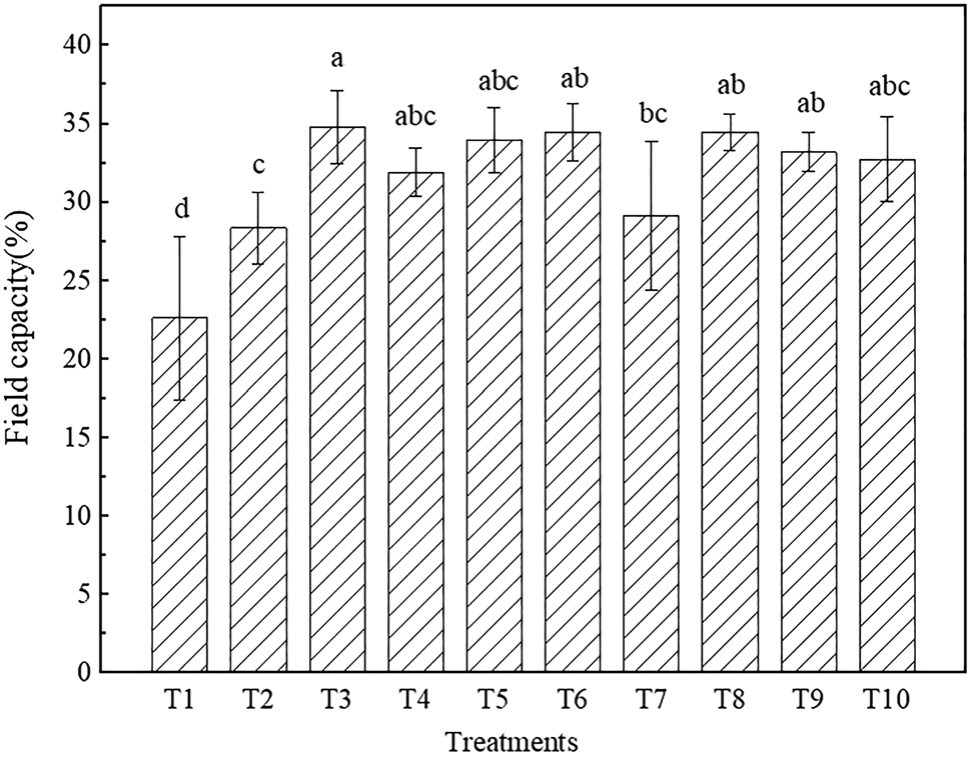
Field capacity under the different treatments (the different letters denote significant differences at the 0.05 level).

The field capacity data are shown in Fig. 5, and the field capacity significantly differed among the different treatments (P < 0.05). Generally, the field capacity ranged from 22.61% to 34.77% and was ranked as T1 < T2 < T7 < T4 < T10 < T5 < T9 < T6 < T8 < T3. T1 exhibited the smallest soil thickness (only 25 cm) and the lowest field capacity. The field capacity under T4 was 12.59% higher than that under T2, and the field capacity under T8 was 18.14% higher than that under T7. The topsoil under T3 was cinnamon soil (b), and the field capacity was 22.65% higher than that under T2, whose topsoil comprised cinnamon soil (a). The field capacity under T3 was 19.20% higher than that under T7, and the value under T5 was 2.26% higher than that under T9. Compared to T2, the field capacities of T6, T5 and T10 were significantly higher, by 21.54%, 19.72% and 15.52%, respectively. Mixing iron tailings into topsoil could improve the field capacity. The field capacity of cinnamon soil (b) was higher than that of cinnamon soil (a). The field capacity obtained by placing iron tailings below topsoil was higher than that obtained by placing red clay below topsoil. Increasing the soil thickness could improve the field capacity.

#### Soil water content

**Figure 6:**
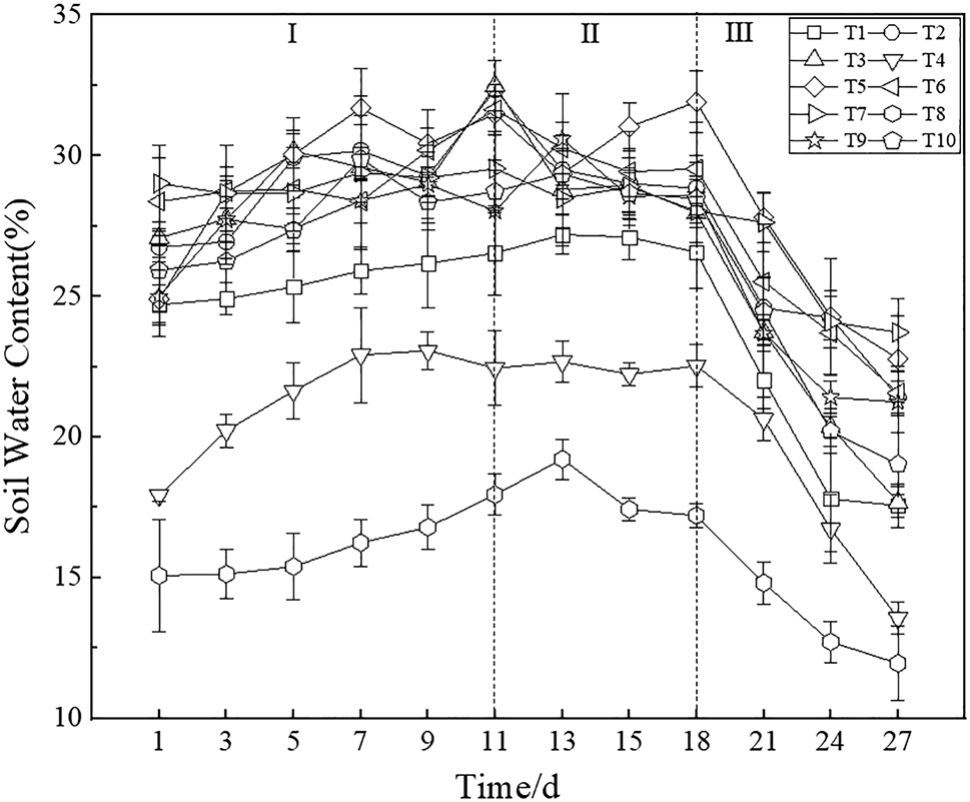
Soil water content under the different treatments (I, II, and III indicate the three stages of soil water content change).

The soil water content under the different treatments during maize growth was monitored for one month, and the results are shown in Fig. 6. Overall, the soil water content revealed fluctuating changes. At the first stage (Days 1 to 11), all treatments exhibited a high soil water content, and the trend gradually increased. At the second stage (Days 11 to 18), the soil water content under all treatments basically reached the highest value and tended to remain stable. In addition, the duration of the second stage was shorter than that of the first stage. At the third stage (Days 18 to 27), the soil water content under all treatments gradually decreased and reached a minimum on Day 27. At the stable stage, the soil water content was ranked as T8 < T4 < T1 < T7 < T3 < T10 < T2 < T6 < T9 < T5. The soil water content under T5 was 11.53% higher than that under T9, and the value under T3 was almost the same as that under T7. The soil water content under T6 was 0.65% higher than that under T2, which reflected that the thickness of the iron tailings layer could affect the topsoil water content.

#### Soil EC

**Figure 7:**
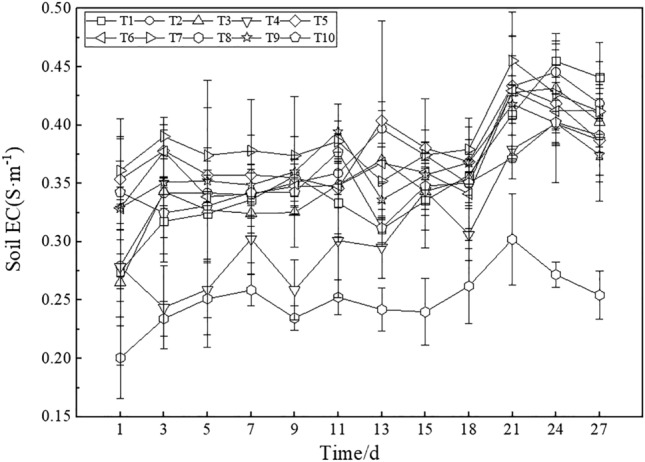
Soil EC under the different treatments.

A moisture teller was used to monitor the soil EC under each treatment during maize growth for one month, and the results are shown in Fig. 7. The soil EC of the topsoil layer under each treatment indicated an overall fluctuating trend with the value increasing before Day 21 and then decreasing thereafter. Similar to the soil water content, the soil EC under T8 was the lowest at each time, with a value ranging from 0.20 to 0.30 S·m^−1^, followed by T4, with values ranging from 0.24 to 0.40 S m^−1^. Overall, the soil EC was ranked as T8 < T4 < T10 < T1 < T3 < T9 < T6 < T2 < T5 < T7. Normal growth and development of crops require soil nonsalinization, in which the soil EC value should be lower than 2 S m^−1^^44^. The maximum EC of the topsoil under all treatments was 0.46 S m^−1^, which is far lower than the threshold limit. Therefore, the soil EC under each treatment indicated suitable growth and development conditions for crops.

### Crop growth and yield

#### Plant height

**Figure 8:**
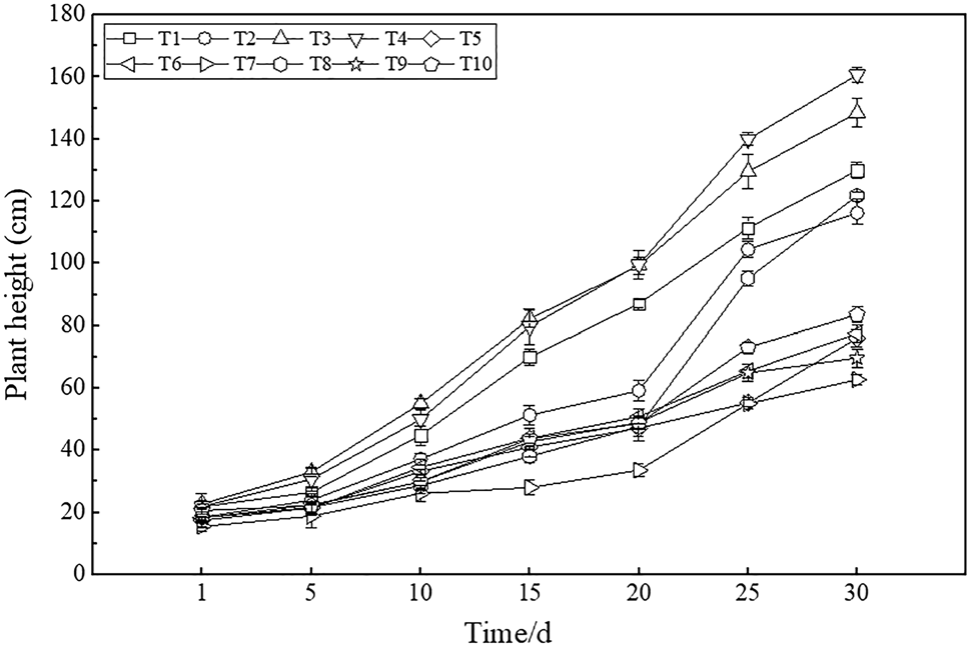
Plant height under the different treatments.

The plant height of maize was measured for one month during the maize growing period. As shown in Fig. 8, the plant height of maize revealed an obvious upward trend during the monitoring period. Overall, the plant height under all treatments was ranked as T7 < T9 < T5 < T6 < T10 < T2 < T8 < T1 < T3 < T4. The plant height under T4 was 38.28% greater than that under T2, and the value under T8 was 94.35% larger than that under T7. The plant height under T3 was 136.82% greater than that under T7, and the value under T5 was 9.13% larger than that under T9. Compared to T2, the plant heights under T6, T5 and T10 were significantly smaller, by 49.91%, 52.83% and 39.11%, respectively.

#### Stem thickness

**Figure 9:**
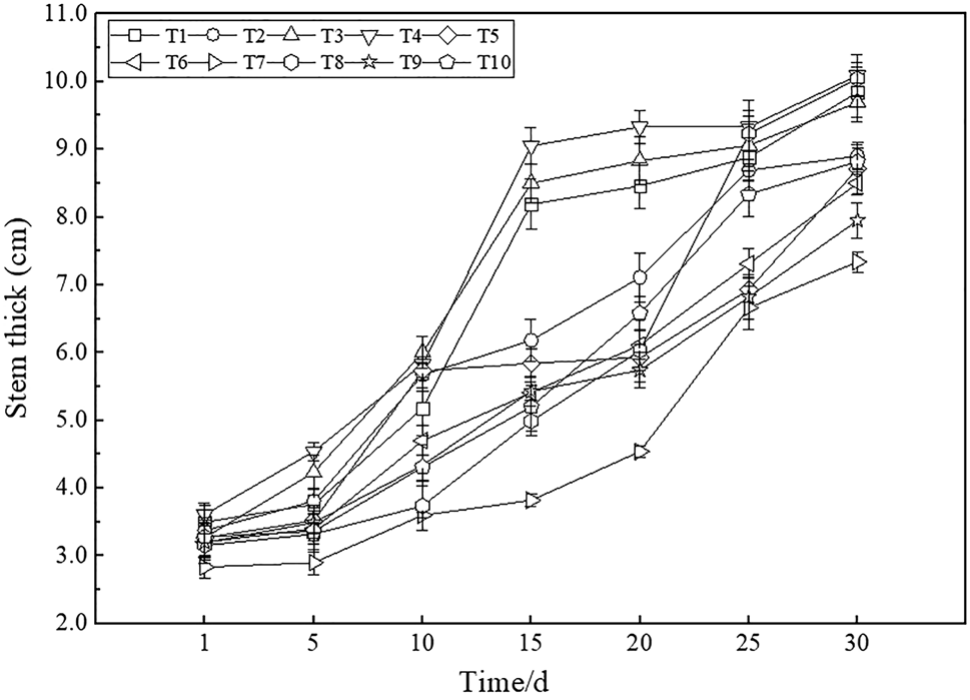
Stem thickness under the different treatments.

The stem thickness of maize was determined while measuring the plant height, and the results are shown in Fig. 9. The stem thickness of maize also exhibited an obvious upward trend during the monitoring period. Based on the obtained data, the plant height under all treatments was ranked as T7 < T9 < T5 < T6 < T10 < T2 < T3 < T1 < T8 < T4, and the variation trend was consistent with that of the plant height. The stem thickness under T4 was 13.37% larger than that under T2, and that under T8 was 36.78% larger than that under T7. The stem thickness under T3 was 32.02% larger than that under T7, and the value under T5 was 9.56% larger than that under T9. Compared to T2, the stem thicknesses under T6, T5 and T10 were significantly smaller, by 4.91%, 2.18% and 0.91%, respectively.

#### Dry weight

**Figure 10:**
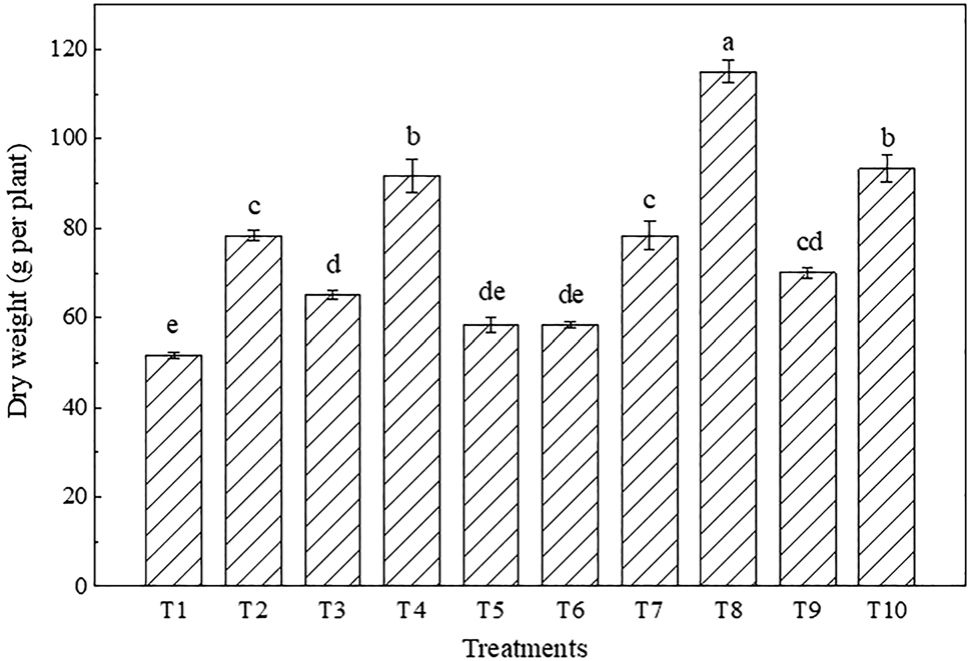
Dry weight under the different treatments (the different letters denote significant differences at the 0.05 level).

The dry weight data of maize are shown in Fig. 10, and the dry weight significantly differed among the different treatments (P < 0.05). Generally, the dry weight was ranked as T1 < T5 < T6 < T3 < T9 < T7 < T2 < T4 < T10 < T8. T1 achieved the minimum dry weight (only 51.67 g per plant). The dry weight under T4 was 17.03% higher than that under T2, and the value under T8 was 46.81% higher than that under T7. The dry weight under T3 was 20.51% lower than that under T7, and the value under T5 was 20.0% lower than that under T9. Compared to T2, the dry weights under T6 and T5 were all 34.29% lower, but that under T10 was 19.15% higher. The application of a mixed 5-cm iron tailings-topsoil layer could facilitate dry material accumulation in crops. Placing red clay below topsoil was more conducive to dry matter accumulation than was placing iron tailings.

#### Crop yields

**Figure 11:**
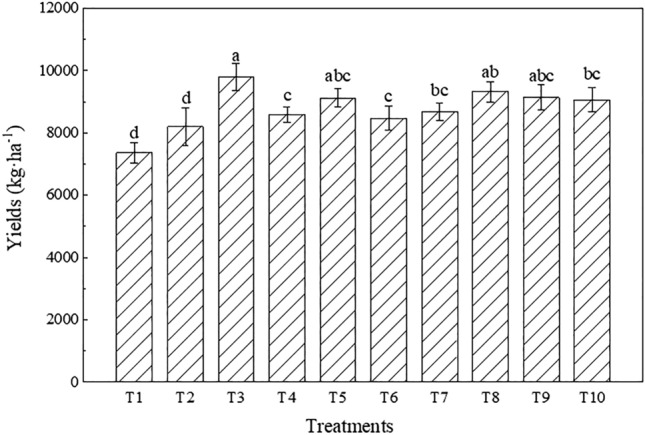
Crop yields under the different treatments (the different letters denote significant differences at the 0.05 level).

Maize was harvested in October 2020. The crop yields under each treatment are shown in Fig. 11. Overall, the maize yields under all treatments were higher than 7, 000 kg·ha^−1^ and indicated no significant differences among the different treatments (P < 0.05), while the crop yields could be ranked as T1 < T2 < T6 < T4 < T7 < T10 < T5 < T9 < T8 < T3. T3 attained the maximum yield (9,793.92 kg·ha^−1^). The yield under T4 was 19.24% higher than that under T2, and the value under T8 was 7.32% higher than that under T7. The yield under T3 was 12.69% higher than that under T7, and the crop yield under T5 was basically the same as that under T9. Compared to the T2 treatment, the yields under the T6, T5 and T10 treatments were all higher, by 17.66%, 26.72% and 25.83%, respectively.

## Discussion

### Feasibility of farmland construction with iron tailings and its influence mechanism on soil properties and crops

The theory of soil-forming factors posits that soil is the product of multiple natural factors, such as biology, climate, parent material, topography, and time, as well as human activities^50^. In this study, based on the theory of soil-forming factors and reconstruction of soil parent material, different farmland soil profiles were constructed using solid waste materials (waste rocks, gravel particles, soil stripping materials, and iron tailings) generated during mining as soil substitute materials. The entire reclamation project was completed in two months, and maize was planted in the same year. The maize growth level was basically consistent with that in local normal farmland, and there was no contamination in the reclaimed farmland plots, which indicate the feasibility of constructing farmland involving large amounts of iron tailings.

Agriculture in the semiarid area of western Liaoning is supported by natural rainfall, and atmospheric precipitation is the main source of soil moisture^51^. Improving the soil storage capacity above that attributable to atmospheric precipitation is the key to agricultural production in western Liaoning. Due to the clayey soil texture of native soil in western Liaoning, its water retention capacity is poor. Therefore, the main purpose of using iron tailings to reclaim farmland is to obtain a certain thickness of the iron tailings layer below topsoil as a water-retaining layer in farmland and improve the water-retaining capacity. The field capacity is frequently used to measure the water retention capacity of soil, which exhibits significance in guiding agricultural production and irrigation^52,53^. The results of the field capacity and soil water content in the experiment revealed that the field capacity under all treatments entailing the placement of iron tailings below topsoil was higher than that under the treatments entailing the placement of red clay below topsoil. In addition, increasing the filling thickness of iron tailings could effectively improve the field capacity. The reason is that the specific surface area is closely related to the field capacity, and the higher the specific surface area is, the greater the ability to accept water molecules and the greater the ability to conserve water^53,54^. Iron tailings are mostly irregular granular materials^55^, and their specific surface area is higher than that of clay particles, resulting in an increase in the field capacity. With increasing filling thickness of iron tailings, the water retention capacity increased. Regarding the soil water content, variation may be affected by regional rainfall. At the early stage of monitoring (1st–18th days), i.e., in early and middle July, regional rainfall occurred frequently, and the soil water content increased and stabilized. However, during the late monitoring period (18th–27th days), i.e., in late July, regional rainfall occurred less frequently, and evaporation was high, resulting in a significant decrease in the soil water content, which eventually stabilized. Moreover, the soil water content obtained by placing iron tailings below topsoil was equivalent to or even higher than that obtained by placing red clay. Although the moisture content in regional dry discharge iron tailings is low, only 8.9%, due to the satisfactory water retention capacity of its irregular particles, iron tailings could effectively store rainfall and provide water for topsoil during regional droughts, so that the moisture content in reclaimed soil was higher than that in regional normal farmland soil. The above fully demonstrates that when a large amount of iron tailings is used to construct farmland in the semiarid area of western Liaoning, placing an iron tailings layer of certain thickness instead of soil below topsoil as a water-retaining layer could represent a suitable way to effectively improve the moisture characteristics of reclaimed soil.

An increase in soil moisture may lead to an increase in soil salinity. The processes of salt transport with water and salt removal by water vividly describe the influence of water on soil salinity movement^56^. The placement of iron tailings below the topsoil layer as a water-retaining layer may increase the content of water-soluble salts in soil and limit seed germination and crop growth when improving soil moisture in the region. It has been widely reported that the soil EC is an index for soil water-soluble salt determination, which is an important soil property^44^. The dynamic monitoring results of the soil EC indicated that EC exhibited an overall upward trend from Days 1 to 21 but gradually decreased thereafter. The whole process is consistent with the change in the soil water content. The soil EC under the treatments involving the placement of iron tailings below topsoil was higher than that under the treatments involving the placement of red clay below topsoil during the monitoring period, indicating that placing iron tailings below topsoil could increase the soil EC. The EC of iron tailings is approximately 30% lower than that of topsoil because the moisture content in iron tailings (8.9%) is lower than that in topsoil (approximately 48%). However, placing iron tailings below topsoil could increase the topsoil EC because when iron tailings are used as a water-retaining layer, the resulting moisture content is higher than that in regional native soil, and when there occurs high evaporation in the region and a lack of rainfall, the iron tailings layer could provide more moisture for topsoil so that more salt could become concentrated in the topsoil layer. Normal growth and development of crops require soil nonsalinization, in which the soil EC value should be lower than 2 S·m^−1^^44^. Although placing iron tailings below topsoil could increase the soil EC, the dynamic monitoring results indicated that the maximum soil EC under each iron tailings filling structure treatment was 0.43 S m^−1^, which is far below the salt tolerance limit of crop growth. Therefore, the use of large amounts of iron tailings instead of soil for filling below topsoil as a water-retaining layer will not result in salt injury to crops, which further proves that the use of large amounts of iron tailings in farmland construction is feasible.

Crop growth and yield are important indicators to determine whether the performance of reconstructed farmland is satisfactory. Soil drought and water shortages are important factors restricting crop growth and leading to low yields in western Liaoning^57^. Soil drought reduces the water use efficiency, destroys photosynthetic organs, hinders crop metabolism and root growth, and directly affects the crop chlorophyll content^58^. Maize is the dominant crop in western Liaoning, its roots are mainly concentrated in the 0–40 cm soil layer, and the soil moisture content in the root layer directly affects crop root development^59^. The results demonstrated that the plant height and stem thickness of maize under the treatments involving the placement of iron tailings below topsoil were higher than those under the treatments involving the placement of red clay below topsoil, which suggested that the use of iron tailings instead of soil below topsoil could effectively promote maize growth. Compared to normal farmland, the plant height of maize grown in iron tailings-filled farmland was 9.13% greater, and the stem thickness was 9.56% larger at maturity. The reason is that the water-retaining layer comprising iron tailings could effectively improve the soil water content, thereby enhancing the water utilization rate so that maize would not lack moisture during growth and could grow fast. In terms of the maize yield, the value under T3 was 12.69% higher than that under T7, and the maize yields under T5 and T9 were basically the same. The maize yield in normal farmland in western Liaoning is approximately 9,000 kg·ha^−1^^60^. The yields under T3, T5 and T10 all exceeded 9,000 kg·ha^−1^, which is higher than that in local normal farmland, indicating that the maize yield in farmland constructed with iron tailings was equivalent to or even better than that in regional normal farmland during the year of construction. compared to T2 (20-cm iron tailings layer below a 20-cm topsoil layer), the yields under T5 (20-cm iron tailings layer below a 30-cm topsoil layer) and T6 (30-cm iron tailings layer below a 20-cm topsoil layer) were 26.72% and 17.66% higher, respectively, indicating that the increase in the thickness of the constructing layer could improve the maize yield to a certain extent. This may occur because the increase in the thickness of the constructing layer could effectively improve the field capacity and moisture content, effectively enhance the growth of maize, and provide the moisture needed for the formation of maize grains during the grain-filling period, thereby increasing the maize yield. Thus, from the perspective of crop growth and yield, it is feasible to use a large amount of iron tailings instead of soil to construct a farmland water-retaining layer, which could promote maize growth and effectively improve the maize yield.

### Influence mechanism of farmland topsoil mixed with iron tailings on the soil properties and crops

Our research group previously proposed that the application of a 5-cm iron tailings layer atop topsoil in farmland could improve the soil texture and soil moisture characteristics while increasing the soil thickness. Therefore, we applied this technique in the process of using a large amount of iron tailings to construct farmland for further verification. The soil texture of the original farmland soil in western Liaoning is dense, belonging to silty clay loam, which seriously affects crop growth^60^. Mixing sand in clay soil is an effective way to improve the clayey soil texture^61^, and as expected, the results in this study supported this finding. The bulk density is one of the important indices reflecting the soil texture, which is closely related to soil and agricultural ecosystems and notably influences soil thermal, hydraulic and mechanical properties^62,63^. Our results indicated that the bulk density under the treatment with a 5-cm mixed iron tailings-topsoil layer was 3.08% lower than that under the treatment with no iron tailings mixed into topsoil, which indicates that the application of a 5-cm iron tailings layer atop topsoil could effectively reduce the soil bulk density. The reason is that although the bulk density of iron tailings (1.53 g·cm^−3^) is higher than that of regional stripped topsoil (1.39 or 1.41 g·cm^−3^) (Table 1), iron tailings comprise irregular particles, and the sand content is high. After application to topsoil, iron tailings are fully mixed with the topsoil via tillage, which effectively alters the mechanical composition of the soil layer and thus changes the soil porosity. The bulk density is usually related to the soil porosity^64^. An increase in the sand content could yield a loose and porous soil environment and increase the soil porosity, thereby reducing the bulk density and effectively improving the texture of the native clayey soil.

Changes in the soil texture could lead to changes in soil hydrothermal conditions, thereby affecting the soil quality and crop growth and yield. In our study, the field capacity, soil water content, maize plant height, stem thickness and yield obtained by placing a 5-cm iron tailings layer atop topsoil were all better than those obtained by not mixing iron tailings into topsoil. The reason is that the application of iron tailings to topsoil followed by plowing and mixing could increase the soil porosity and decrease the bulk density. Moreover, the bulk density and soil porosity greatly influence the soil water content^65^. An increase in the soil porosity could enhance the distribution of soil pores, expand the soil storage space, store more water in soil pores, and effectively improve the soil water holding capacity. Although the application of a 5-cm iron tailings layer atop topsoil increased the soil moisture content, resulting in an increase in the soil EC to a certain extent, its value was far below the maximum salt tolerance limit of crop growth and would not result in salt injury to crops. Soil texture improvement and soil moisture content increase could effectively promote maize growth, and the plant height and stem thickness of maize during the growth period were 9.13% and 9.56% greater, respectively, than those under the treatments without iron tailings application. At the maize grain-filling stage, the increase in soil moisture was beneficial to the formation of maize grains, and the yield of maize was 12.69% higher than that under the treatments without iron tailings application. These results are also consistent with the findings of Yang^38^ and Ji et al.^66^, who reported that applying iron tailings to topsoil significantly promoted crop growth. Other studies have also reported that restoration efforts in arid ecosystems should focus on increasing the sand content in soils near the surface to reduce the evaporative water loss and improve the soil quality and crop health^67^.

### Benefits and limitations of constructing farmland with large amounts of iron tailings

The selection of a reasonable construction scheme based on experiments using solid waste materials produced in mining processes as soil substitute materials is of great significance for land reclamation in mining areas with scarce soil resources. First, this allows the problem of poor vegetation growth due to an insufficient thickness of the overlying soil in these areas to be solved. Second, the cost to mining enterprises of purchasing reclaimed soil is greatly reduced, as soil reconstruction using substitute materials can save more than 50% of the available soil; for example, assuming a soil cover thickness of 30 cm, the total amount of soil needed is less than 1500 m^3^ per hectare, and if the local price of topsoil is approximately 30 CNY ·m^−3^, then, in this case, the reclamation investment cost can be reduced by 45,000 CNY per hectare^50^. While saving many guest soil sources, secondary land damage is reduced. Finally, the problem of the disposal of solid waste generated in the mining process can be solved. Agricultural resource utilization of iron tailings, used for medium- and low-yield field improvement and reclamation, not only consumes large amounts of beneficiation iron tailings accumulated in inventories but also prevents land occupation and contamination problems attributed to iron tailings accumulation and improves medium- and low-yield fields, thereby greatly protecting soil resources. It can thus be observed that using solid waste as a soil substitute material can provide certain economic and ecological benefits.

The study preliminarily analyzed the physical and hydrological characteristics of soil under different construction measures in experimental plots using iron tailings to replace soil for farmland construction and revealed that it was feasible to use iron ore waste materials such as iron tailings and waste rocks for soil and farmland construction enhancement. Since the experimental demonstration was only conducted for one year, the relevant experimental observation data are insufficient. In the future, it is necessary to establish long-term experimental monitoring campaigns, further study the influence of various factors on the soil structure, continue to optimize the best structural measures, improve the application effect of this technology, and promote its application accordingly.

Iron tailings should be popularized and applied according to local conditions. At present, most iron tailings are used as fertilizer substrates, and their consumption is low. The use of iron tailings to improve barren soil and construct farmland prevents the problem of passive tailings pond reclamation due to the notable accumulation of mineral processing tailings in the past, which is a suitable method for resource utilization of iron tailings and can provide broad application prospects. However, due to the limitations of the obtained monitoring data and other aspects, this study only preliminarily verified the soil characteristics and effects of the application level of iron tailings in Jianping County, and more comprehensive research and analysis and evaluation studies should be conducted in the future. In addition, mining waste rock and soil fill pit and reclamation projects are underway, which can improve the entire iron ore waste agricultural resource utilization technique, forming a complete set of technical systems.

## Conclusions

Aiming at the problem of iron ore waste resource utilization and lack of soil for soil reconstruction, we proposed the technology of using a large amount of iron tailings to reconstruct reclaimed farmland soil. The experimental application of various treatments to newly reclaimed farmland revealed that iron tailings could be considered effective soil substitute materials to construct farmland and improve the soil texture and water content. Moreover, the maize growth in the experimental plots was normal, and the crop yields were close to or even higher than those in local normal farmland. Iron tailings could be recommended as a feasible and harmless material for farmland construction because of the advantages in improving the soil quality and crop yield.

For relatively barren local topsoil layers, the application of a 5-cm mixed iron tailings-topsoil layer could effectively improve the textural characteristics of clayey soil in western Liaoning, increase the soil water capacity, and enhance soil moisture, atmosphere, and thermal conditions, which was conducive to maize growth and achieved the purpose of generating a high yield.

Given the semiarid climatic conditions in western Liaoning, solid waste resulting from iron ore mining could be used as farmland construction material to establish a water-retaining layer below topsoil, and satisfactory results could be obtained. At the same time, the accumulated waste rocks, topsoil stripping materials, iron tailings, and other waste materials could be consumed, resulting in higher economic and ecological benefits, which represents a suitable way for agricultural resource utilization of iron ore waste materials.

## Data Availability

All data generated or analysed during this study are included in this published article.
